# Supplementary material for: A pilot cost-benefit analysis of a children’s spectacle reimbursement scheme: Evidence for Including children’s spectacles in Mongolia’s Social Health Insurance
Source: PLoS One. 2022 Aug 15;17(8):e0273032. doi: 10.1371/journal.pone.0273032 (PMC9377584; doi:10.1371/journal.pone.0273032)
Supplement: Attachment — Submitted filename: Response to Reviewers_CBA Mongolia_20220502.docx [file pone.0273032.s004.docx]

**PONE-D-21-24356R1**

**A Pilot Cost-Benefit Analysis of a Children’s Spectacle Reimbursement Scheme: Evidence for Including Children’s Spectacles in Mongolia’s Social Health Insurance**

Comments to the Author

Review Comments to the Author

**Reviewer #1:**

It is good to read the revised manuscript with acknowledgement of the limitations on the generalisability of the results due to small sample size and no income threshold data to relate the validity of the findings. As indicated (with $19 benefit for RMS), the results suggest relatively high WTP of Mongolians despite 90% RE children (relating half suitable for ready-made) being possibly uncorrected. In addition to this, those with RE are more likely to pay less than those without, is certainly question of investigation. As concluded, the full-scale study with greater sample size and proper selection of participants, will hopefully contribute to purposing the structure of reimbursement for spectacle.

1. Unlike this study (spectacle reimbursement scheme), previous WTP studies contribute/suggest for “spectacle cost-subsidisation scheme”. You may discuss the difference and highlight the SMI features and focus. Importantly, is this meant for RMS, custom-made or both? The question and response are dependent to the specific product and accordingly relate the scheme. Ideally, WTP question is to base on the focus or the need; for ready-made or custom-made. If relevant and useful, add to highlight.

**Response: Thank you for suggesting this. We added this into our Discussions: *“The observation of Cambodian respondents in offering lower WTP can be explained by the following reasons. Firstly, the proposed scheme in Mongolia was to include spectacle provision through the Social Health Insurance which will not incur any payment at the service points, while in Cambodia, the proposed cross-subsidisation scheme will require parents to pay a nominal amount. Secondly, the difference may be due to a higher gross domestic product per capita in Mongolia than Cambodia (US$4,339 versus US$1,643).[35,36] Lastly, our study uses additional annual taxes as the payment vehicle, while in Cambodia, the payment was through out-of-pocket expenses.”***

**We recognised the limitation of not specifying which types of spectacles (ready-made or custom-made) that the tax payer would be willing to pay. However, we chose to use mix spectacles (US$15) as the spectacle cost because i) one in two Mongolian children who had refractive error can be corrected effectively from a pair of inexpensive, ready-made spectacles, and ii) to reflect the practical situation where there will be need to have a combination of both ready-made and custom-made spectacles to cover the refractive needs of the children. *“******Based on a dataset obtained from an outreach vision screening programme initiated by an international eye NGO, one in two Mongolian children who had refractive error can be corrected effectively from a pair of inexpensive, ready-made spectacles. Therefore,*** ***the costs of ready-made spectacles and custom spectacles were used to construct the cost of a mixed offering to reflect the need for a combination of both spectacle types to address the refractive needs of the children.”* We included this content in the Methods section.**

**We also highlighted the following in the Limitations *“In addition, employing the costs of ready-made spectacles and custom spectacles to construct the cost of a mixed offering may have also confounded the results, but we felt it was necessary to reflect the local refractive needs.”***

2. Discussion third paragraph - Unlike this study, reference no 34 do not have a compare group. The Cambodia study included only parents of children with RE. Please correct ‘an eye disorder’ with ‘refractive error’. About 53% parents were willing to pay $17.50 (standard price of custom-made spectacle) or more.

**Response: Thank you for spotting this error. We have amended the sentence accordingly.**

3. Reason for “Those with RE had low WTP compared to without” is simply unanswerable in given situation. The listed potential reasons (different appreciation and income) do not quite relate or suit. I suggest delete and simply indicate the need for clarification /verification through full-scale study.

**Response: Thank you for highlighting this. We agreed that findings of “those with RE had low WTP compared to without” is unanswerable. We also agreed that there is a need for clarification/verification through full-scale study, as added to the Discussion *“This should be examined in further research with a larger sample and where details of income are collected”*.**

4. In the concluding statements, it is better to state clearly about the scheme that it is the proposed or potential spectacle reimbursement scheme under SMI.

**Response: Thank you for highlighting this. In the conclusion, we made clear statement that, *“Our preliminary findings suggest that there is potential to include children’s spectacles into the existing Social Health Insurance. However, further research with larger sample size is needed to confirm this.”***

**Reviewer #2: (No Response)**
