# Supplementary material for: A pilot cost-benefit analysis of a children’s spectacle reimbursement scheme: Evidence for Including children’s spectacles in Mongolia’s Social Health Insurance
Source: PLoS One. 2022 Aug 15;17(8):e0273032. doi: 10.1371/journal.pone.0273032 (PMC9377584; doi:10.1371/journal.pone.0273032)
Supplement: Attachment — Submitted filename: WTP Mongolia Reviewer comments.docx [file pone.0273032.s003.docx]

It is good to read the revised manuscript with acknowledgement of the limitations on the generalisability of the results due to small sample size and no income threshold data to relate the validity of the findings. As indicated (with $19 benefit for RMS), the results suggest relatively high WTP of Mongolians despite 90% RE children (relating half suitable for ready-made) being possibly uncorrected. In addition to this, those with RE are more likely to pay less than those without, is certainly question of investigation. As concluded, the full-scale study with greater sample size and proper selection of participants, will hopefully contribute to purposing the structure of reimbursement for spectacle.

Unlike this study (spectacle reimbursement scheme), previous WTP studies contribute/suggest for “spectacle cost-subsidisation scheme”. You may discuss the difference and highlight the SMI features and focus. Importantly, is this meant for RMS, custom-made or both? The question and response are dependent to the specific product and accordingly relate the scheme. Ideally, WTP question is to base on the focus or the need; for ready-made or custom-made. If relevant and useful, add to highlight.

Discussion third paragraph - Unlike this study, reference no 34 do not have a compare group. The Cambodia study included only parents of children with RE. Please correct ‘an eye disorder’ with ‘refractive error’. About 53% parents were willing to pay $17.50 (standard price of custom-made spectacle) or more.

Reason for “Those with RE had low WTP compared to without” is simply unanswerable in given situation. The listed potential reasons (different appreciation and income) do not quite relate or suit. I suggest delete and simply indicate the need for clarification /verification through full-scale study.

In the concluding statements, it is better to state clearly about the scheme that it is the proposed or potential spectacle reimbursement scheme under SMI.
